# Recent Advances in Nanotechnology-Based Strategies for Bone Tuberculosis Management

**DOI:** 10.3390/ph17020170

**Published:** 2024-01-29

**Authors:** Yuanrui Luo, Hongwei Chen, Hua Chen, Peng Xiu, Jiancheng Zeng, Yueming Song, Tao Li

**Affiliations:** Department of Orthopedic Surgery and Orthopedic Research Institute, West China Hospital, Sichuan University, No. 37 Guo Xue Xiang, Chengdu 610041, China; luoyuanrui2021@163.com (Y.L.); hongweichenhx@163.com (H.C.); hxchenhua1987@hotmail.com (H.C.); xiup200303@126.com (P.X.); tomzeng5@163.com (J.Z.); hx_sym@163.com (Y.S.)

**Keywords:** bone tuberculosis, nanoscale, drug delivery systems, treatment

## Abstract

Bone tuberculosis, an extrapulmonary manifestation of tuberculosis, presents unique treatment challenges, including its insidious onset and complex pathology. While advancements in anti-tubercular therapy have been made, the efficacy is often limited by difficulties in achieving targeted drug concentrations and avoiding systemic toxicity. The intricate bone structure and presence of granulomas further impede effective drug delivery. Nano-drug delivery systems have emerged as a promising alternative, offering the enhanced targeting of anti-tubercular drugs. These systems, characterized by their minute size and adaptable surface properties, can be tailored to improve drug solubility, stability, and bioavailability, while also responding to specific stimuli within the bone TB microenvironment for controlled drug release. Nano-drug delivery systems can encapsulate drugs for precise delivery to the infection site. A significant innovation is their integration with prosthetics or biomaterials, which aids in both drug delivery and bone reconstruction, addressing the infection and its osteological consequences. This review provides a comprehensive overview of the pathophysiology of bone tuberculosis and its current treatments, emphasizing their limitations. It then delves into the advancements in nano-drug delivery systems, discussing their design, functionality, and role in bone TB therapy. The review assesses their potential in preclinical research, particularly in targeted drug delivery, treatment efficacy, and a reduction of side effects. Finally, it highlights the transformative promise of nanotechnology in bone TB treatments and suggests future research directions in this evolving field.

## 1. Introduction

Tuberculosis (TB) stands as one of humanity’s oldest adversaries in the realm of infectious diseases, with its legacy tracing back to spinal anomalies observed in ancient mummies around 9000 BC [1,2]. Globally, TB remains a pervasive health challenge, infecting an estimated 2 billion people. While a majority harbor a latent infection, approximately 5–15% develop active symptoms, and 1.4 million people die to this disease every year [3,4,5].

TB has historically been linked with socio-economic disparities, more frequently impacting individuals with compromised health or living in conditions that foster its spread, such as overcrowded living spaces. The disease significantly affects vulnerable groups, including the elderly, diabetics, smokers, and individuals with compromised immune systems, such as those with HIV, which dramatically increases the risk of TB [6,7,8].

The incidence of bone TB, a form of extrapulmonary TB, is not precisely known. However, it is understood that a significant portion of TB infections manifest outside the lungs, with skeletal TB occurring in about 10% of those with active pulmonary TB [9]. The primary causative agent, Mycobacterium tuberculosis, typically leads to an infection in the spine and large joints due to their abundant blood supply. The spine is the most frequently affected skeletal site, representing the majority of skeletal TB cases [8,10,11].

Bone TB typically presents with insidious onset and non-specific symptoms, making an early diagnosis challenging [12]. Patients may experience localized pain, swelling, and stiffness in the affected area, often accompanied by systemic symptoms, such as fever, night sweats, and weight loss. As the disease progresses, more severe signs, such as spinal deformities, neurological deficits due to spinal cord compression, or joint dysfunction, can occur [13,14,15,16]. The diagnosis of bone TB relies on a combination of clinical evaluation, imaging techniques such as X-rays and MRI, and laboratory tests, including biopsies and microbiological cultures [17,18,19,20]. However, the similarity of its symptoms to other musculoskeletal conditions, along with the slow-growing nature of the causative bacteria, often leads to diagnostic delays and challenges.

Traditional treatment strategies combine pharmacological and surgical interventions [21,22]. However, the effectiveness of standard anti-TB drugs is compromised by inadequate bone penetration, systemic side effects, and the rising incidence of drug-resistant TB strains, highlighting the need for innovative treatment methodologies [23,24].

Nanotechnology, manipulating materials at an atomic or molecular scale [25], offers transformative prospects in medical treatments, particularly for bone TB. It enables targeted drug delivery, improved drug penetration, and localized treatment [26]. Nanoparticles can be tailored to deliver therapeutics directly to infected bone tissues, enhancing treatment efficacy and minimizing systemic toxicity [27,28]. Beyond drug delivery, the role of nanotechnology is multifaceted in tissue engineering, contributing significantly to advancements in various areas, including but not limited to bone tissue engineering, which is particularly relevant for regenerating TB-affected bone. Innovations include bone scaffolds and nanocomposites that support bone growth and recovery [29,30,31,32].

This review explores the intricacies of bone TB and the groundbreaking solutions provided by nanotechnology. We synthesize the latest advancements and knowledge, offering a comprehensive perspective on how nanotechnology could revolutionize the treatment landscape for bone TB.

## 2. Pathophysiology of Bone TB

### 2.1. Primary Infection, Latency, and Reactivation

**Figure 1:**
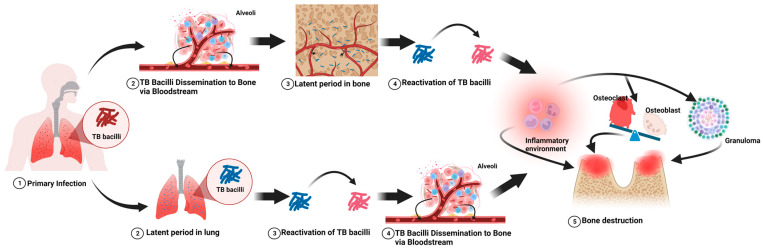
The main pathophysiological process of tuberculosis-induced bone destruction.

Bone TB generally stems from a primary pulmonary infection (Figure 1) [33,34]. Inhaled Mycobacterium TB bacteria settle in lung alveoli, where the immune response forms tubercles, encapsulating the bacteria and leading to a latent TB infection (LTBI). During LTBI, individuals are asymptomatic, carrying the bacteria within granulomas. Various factors such as immune status, genetics, and environmental conditions influence the latency period. Reactivation of TB can occur with a weakened immune system due to conditions such as HIV, stress, or malnutrition, leading to the activation and spread of the bacteria [34]. This spread can manifest in two ways: first, during the initial lung infection, the bacteria might disseminate via the bloodstream to bones, becoming dormant and potentially activating directly within the bone tissue under conducive conditions. Second, a latent infection initially in the lungs can reactivate, spreading to bones through the bloodstream or lymphatic system. This hematogenous spread is concerning for its potential to cause multifocal skeletal involvement, often without evident lung involvement, marking the transition from a localized infection to a systemic condition, which includes skeletal TB.

### 2.2. Predilection for Bone Tissue

Mycobacterium TB, as an obligate aerobe, relies on oxygen-rich environments for survival and replication, which it finds abundantly in bone tissues [35]. The vertebral bodies, part of the axial skeleton, are particularly susceptible due to their rich vascularization, which supplies ample oxygen and nutrients [36,37]. The propensity of Mycobacterium TB for these oxygenated environments is crucial in its pathogenesis, especially in skeletal TB. This preference for well-vascularized bone areas, such as vertebral bodies, underscores the importance of targeted therapeutic approaches. The rich blood supply in these areas not only facilitates bacterial growth but also poses a challenge for drug delivery, as the bacteria can reside deep within the bone, shielded from systemic circulation.

### 2.3. Adhesion to Bone Tissue

Upon reaching the bone tissue, Mycobacterium TB adheres to the bone surface using specific components of its cell wall, such as mycolic acids, which are one long-chain fatty acids that provide Mycobacterium TB with its unique pathogenic capabilities, including the ability to adhere to host tissues. These components interact with receptors on osteoblasts and other bone-forming cells, facilitating bacterial attachment and initiating a series of biological responses [35]. In addition, other cell wall components (lipoarabinomannan and phosphatidylinositol mannosides), surface proteins, interactions with the extracellular matrix, molecular mimicry, biofilm formation, immune evasion, and genetic/epigenetic factors all contribute to the successful adhesion and persistence of Mycobacterium TB in bone tissue [38,39,40,41,42].

### 2.4. Granuloma Formation in Bone Tuberculosis

The immune response to TB in bone tissue involves the recruitment of macrophages to the site of infection. These macrophages attempt to engulf the bacteria but often cannot destroy them, leading to the formation of granulomas [43,44,45]. These granulomas act as a containment strategy by the immune system but also provide a niche where Mycobacterium TB can persist, evading eradication. This persistence within granulomas is a significant factor in the chronicity of bone TB. The granulomatous response, while a defense mechanism, can inadvertently contribute to bone damage through chronic inflammation and the disruption of normal bone remodeling processes [46].

### 2.5. Bone Destruction by Tuberculosis

Bone damage in TB results from a combination of bacterial proliferation, granuloma formation, and chronic inflammation. This inflammation disrupts the normal balance between osteoblasts (bone-forming cells) and osteoclasts (bone-resorbing cells), leading to increased bone resorption and the weakening of the bone structure [47]. The persistent inflammation impairs bone repair mechanisms, leading to demineralization and an increased fracture risk [48]. This paradoxical nature of the immune response in TB, essential for containing the infection, can lead to significant bone damage. The immune system’s response to TB, while critical in controlling the infection, can paradoxically exacerbate bone damage [49,50].

## 3. Current Treatment Approaches for Bone Tuberculosis

### 3.1. Antibiotic Regimen Challenges

The primary treatment for bone TB involves a lengthy antibiotic regimen, typically including isoniazid (INH), rifampicin (RFP), pyrazinamide, and ethambutol, over 12 to 18 months, and even some scholars believe that the anti-tuberculosis course should not be less than 18 months [51,52,53,54,55]. This approach effectively controls the infection but faces notable challenges. A significant issue is the ‘first-pass effect’, where orally administered drugs are extensively metabolized in the liver before reaching the bloodstream, diminishing the active drug’s systemic availability. This results in reduced bioavailability and often requires higher doses for therapeutic efficacy, increasing the risk of side effects such as hepatotoxicity and neurotoxicity [56,57]. Achieving adequate drug concentrations at the bone infection site is another challenge that affects treatment effectiveness. The prolonged treatment duration also raises concerns about patient compliance, as long-term therapy can lead to a range of side effects, from mild gastrointestinal issues to severe hepatotoxicity, potentially impacting adherence to the regimen [58,59,60]. The emergence of multidrug-resistant (MDR) and extensively drug-resistant (XDR) Mycobacterium TB strains further complicates treatment, necessitating second-line drugs that are less effective, are more toxic, and require longer durations [61,62] (Table 1).

### 3.2. Surgical Intervention and Bone Reconstruction

Surgical intervention in bone TB is considered when pharmacological therapy is inadequate or complications arise. Surgery aims to remove infected tissue, relieve pain, restore structural stability, and reconstruct bone defects, particularly in cases of significant bone loss or instability [53,54,55]. Various materials and methods are employed, including autografts (patient’s own bone), allografts (donor bone), synthetic substitutes such as hydroxyapatite and tricalcium phosphate, vascularized grafts, and custom 3D-printed implants or titanium mesh cages [63,64,65,66,67,68,69,70,71,72,73]. Each option offers unique advantages and is chosen based on the defect’s location and extent, the patient’s health, and other individual factors.

However, while these materials effectively fill bone defects and restore structural integrity, they have limitations in drug delivery. Traditional materials do not incorporate or they have a limited capacity to load and sustainably release anti-tubercular drugs, playing a primarily structural role without directly combating TB bacteria [74,75]. This highlights the necessity of continued pharmacological treatment alongside structural repairs for effective infection management (Table 2).

## 4. Nanotechnology in Bone Tuberculosis Treatment

In the realm of bone TB treatment, nanotechnology signifies a revolutionary advancement by manipulating materials at a nanoscale for specific medical applications (Table 3). It notably enhances targeted drug delivery to infected bone tissues, thereby significantly improving treatment efficacy [84,85]. This technology is particularly effective when combined with bone scaffolds, addressing both the eradication of a TB infection and the reconstruction of bone lesions. The nanoparticles, ranging from liposomes to polymeric forms, are designed to overcome the limitations of traditional drug formulations, such as poor penetration into bone tissue and systemic side effects. Their capability for targeted delivery ensures a higher drug concentration at the infection site while minimizing the impact on healthy cells, reducing overall toxicity. Additionally, nanotechnology enables a controlled drug release, sustaining therapeutic levels for longer durations and enhancing patient compliance, a crucial aspect in long-term treatments, such as those for bone TB [86,87,88]. This innovative approach also shows promise in combating drug-resistant TB strains, potentially amplifying the potency of existing medications and reducing the necessary dosage and frequency of administration.

### 4.1. Mesoporous Silica Nanoparticles

Mesoporous silica nanoparticles (MSNs) represent a cutting-edge development in the field of nanomedicine, particularly for drug delivery applications. These nanoparticles are distinguished by their unique structure, characterized by pores in the mesoscale range (2–50 nanometers) [94,95,96]. This mesoporous architecture endows MSNs with a high surface area, facilitating a substantial drug-loading capacity. The versatility of MSNs lies in their ability to encapsulate a wide range of therapeutic agents, from small-molecule drugs to larger biomolecules. Crucially, the pore size and surface chemistry of MSNs can be precisely engineered, allowing for a controlled and sustained release of encapsulated drugs. This capability is particularly beneficial in targeting diseases where localized and prolonged drug delivery is needed. Additionally, MSNs are recognized for their biocompatibility and potential for surface modification, making them ideal candidates for targeted drug delivery systems that aim to reduce systemic side effects and enhance treatment efficacy [97,98,99,100]. These systems, as detailed in studies such as those by Zhu et al. [30], have demonstrated significant improvements in delivering antitubercular drugs, such as INH and RFP, directly to the infected bone tissue. Utilizing MSNs in mesoporous composite scaffolds, these studies have achieved a prolonged and localized release of drugs at the bone TB sites, maintaining effective therapeutic concentrations over extended periods and thereby outperforming traditional drug delivery methods.

Further advancements in MSN technology, as explored in other research, such as the study on composite scaffold drug delivery systems for osteoarticular TB therapy, have shown that MSNs can maintain effective drug concentrations for extended durations [29]. This achievement is crucial for reducing long-term organ damage and minimizing systemic side effects, a significant step forward in the localized treatment of bone TB. Additionally, the integration of MSNs into gelatine-based hydrogels within three-dimensional vertebral body scaffolds, as investigated by Yahia et al. [32,89], represents another innovative approach. These nanoscale drug delivery systems, loaded with antimicrobial agents such as RFP and levofloxacin, provide a localized, sustained release of antibiotics. This technique ensures a high therapeutic index at the infection site while substantially reducing the likelihood of systemic adverse reactions.

In summary, these studies collectively highlight the transformative potential of MSNs in enhancing the efficacy and safety of bone TB treatments, offering a more targeted, efficient, and patient-friendly approach to managing this complex disease.

### 4.2. Tetracycline-Modified Nanoparticles

Tetracycline-modified nanoparticles are an innovative development in the field of nanomedicine, especially in drug delivery systems. These nanoparticles are chemically conjugated with tetracycline, a well-known broad-spectrum antibiotic, recognized for its strong affinity towards bone tissue [101,102]. The conjugation with tetracycline significantly enhances the nanoparticles’ ability to selectively target and accumulate in bone tissues. Tetracycline’s molecular structure allows it to bind readily to the calcium present in bone, making it an ideal candidate for targeting bone-related pathologies [103]. When attached to nanoparticles, this attribute is harnessed to direct therapeutic agents specifically to bone tissues. This targeted approach is particularly advantageous for diseases such as bone TB, where the precise delivery of anti-tubercular drugs to the affected bone areas is crucial for effective treatment [104]. The nanoparticles themselves are designed to carry and release these therapeutic agents in a controlled manner. This controlled release is crucial for maintaining effective drug concentrations at the site of infection over an extended period, which is particularly important for the treatment of TB due to the slow-growing nature of Mycobacterium TB. Additionally, the ability to modify the surface of these nanoparticles allows for further functionalization, potentially enhancing their specificity and efficiency in drug delivery. For instance, surface modifications can be made to improve nanoparticle stability, evade the immune system, or facilitate deeper penetration into the infected bone tissues. Moreover, the use of tetracycline-modified nanoparticles in drug delivery helps minimize systemic side effects. Traditional treatments for bone TB often involve high doses of medication, which can lead to adverse effects throughout the body. By concentrating the drug delivery directly at the site of infection, these nanoparticles reduce the likelihood of such systemic side effects, thereby potentially improving patient compliance and the overall safety profile of the treatment.

In the innovative study led by Liang et al. [28], tetracycline-modified nanoparticles (TC-PLGA-PEG NPs) were specially crafted for the targeted delivery of rifapentine, an anti-tubercular drug. This nanoscale drug delivery system was designed to enhance rifapentine concentrations, specifically in bone tissue, while limiting its accumulation in other organs. A notable outcome of this study was the system’s ability to prolong the release of rifapentine in the bloodstream, an essential factor in the treatment of osteoarticular TB, which requires a sustained drug presence at the targeted site for effective therapy. Furthermore, the study demonstrated that these nanoparticles could amplify the anti-TB activity of rifapentine, addressing the significant challenge of treating osteoarticular TB with conventional drug therapies. Importantly, the TC-PLGA-PEG nanoparticles offer the potential to reduce the dosage and frequency required for treating osteoarticular TB, addressing common issues related to severe side effects and the risk of developing multidrug-resistant TB strains inherent in traditional treatments.

### 4.3. Liposomes Nanoparticles

Liposomes, spherical vesicles with a lipid bilayer structure, represent a groundbreaking advancement in nanoscale drug delivery systems. These versatile carriers, typically ranging from 50 to 200 nanometers in size, are unique in their ability to encapsulate a wide range of therapeutic agents, from small molecule drugs to macromolecules, such as proteins and nucleic acids [105,106]. The core structure of liposomes is composed of phospholipids, similar to cell membranes, which endow them with biocompatibility and reduced toxicity. This biomimetic nature facilitates their integration into biological systems, allowing for efficient drug delivery while minimizing immune responses [107,108]. Liposomes can encapsulate both hydrophilic drugs in their aqueous core and hydrophobic drugs within the lipid bilayer, making them uniquely adept at delivering a broad spectrum of therapeutic agents. Their ability to encapsulate anti-tubercular drugs protects these medications from premature degradation in the body, enhancing their stability and efficacy. This encapsulation also ensures a controlled release of the drug, maintaining therapeutic concentrations at the site of infection for extended periods. This controlled release is particularly advantageous in treating bone TB, where prolonged exposure to the drug is necessary to effectively combat the slow-replicating Mycobacterium TB [109,110,111]. Moreover, the surface of liposomes can be modified with targeting ligands or antibodies, allowing for the selective targeting of infected bone tissue. This targeted delivery is crucial in bone TB, where the infection can reside deep within the bone, away from systemic circulation. Targeted liposomes can thus deliver high concentrations of drugs directly to the site of the infection, enhancing treatment efficacy while reducing systemic side effects typically associated with conventional TB therapies. Another significant advantage of liposomes in bone TB treatments is their potential to overcome drug resistance. By delivering high concentrations of drugs directly to the site of the infection, liposomes can effectively combat Mycobacterium TB strains that have developed resistance to standard treatments. This targeted approach not only increases the effectiveness of existing drugs but also opens the possibility of using new drug combinations that may be too toxic for systemic administration [112,113,114]. The flexibility of liposomes extends to their ability to respond to environmental stimuli. For instance, liposomes can be designed to release their payload in response to pH changes, enzymes, or temperature variations, common in infected bone tissues. This responsiveness ensures that the drug is released in a more controlled manner, maximizing its therapeutic impact [115,116].

In a notable study by Liu et al. [85], a liposome-in-hydrogel system was used to encapsulate N′-Dodecanoylisonicotinohydrazide (DINH), a hydrophobic derivative of INH, a first-line antitubercular drug. This system was innovatively designed for targeted bone TB treatment. A localized delivery via this system was found to significantly increase drug concentrations at the infection site, thereby enhancing the therapeutic effectiveness. Its capability to ensure sustained drug release is vital for maintaining consistent therapeutic drug levels and is crucial for ongoing treatment efficacy. Furthermore, this feature allows for less frequent dosing, which could considerably improve patient adherence. Importantly, the study underscored the system’s safety benefit by localizing drug delivery to the infection site and reducing systemic exposure, thus substantially lowering the systemic side effects typically associated with INH therapy.

### 4.4. Poly(Lactide-Co-Glycolide) Nanoparticles

Poly(lactide-co-glycolide) (PLGA), a copolymer used in nanoscale drug delivery systems, is synthesized from two biocompatible and biodegradable constituents: lactic acid and glycolic acid. This copolymer’s significance in medical applications, particularly in targeted drug delivery, lies in its favorable properties, which include biodegradability, biocompatibility, and the ability to facilitate a controlled drug release [117,118]. PLGA degrades in the body through hydrolysis, breaking down into its monomer components, lactic acid, and glycolic acid, which are then metabolized via the Krebs cycle and eliminated from the body as carbon dioxide and water. The degradation rate of PLGA can be tailored by adjusting the ratio of lactide to glycolide; higher lactide content generally leads to slower degradation. In drug delivery, PLGA nanoparticles are designed to encapsulate therapeutic agents efficiently. Their encapsulation capacity is notable, as they can carry a wide range of drugs, including small molecules and macromolecules, such as proteins. Once administered, these nanoparticles gradually release their encapsulated drug over a period that can range from days to several weeks depending on the PLGA composition and particle design. In the context of bone TB, PLGA nanoparticles offer a promising approach for delivering anti-tubercular drugs directly to the infected bone tissue. Their small size allows for penetration into the bone matrix, potentially overcoming one of the significant challenges in bone TB treatment: the effective delivery of drugs to the site of infection. The ability to control the release rate of the encapsulated drug means that therapeutic levels can be maintained at the infection site for prolonged periods, which is vital given the slow replication rate of Mycobacterium TB and the lengthy treatment regimen required for TB [119,120,121]. Further, surface modifications of PLGA nanoparticles can enhance targeting to specific tissues or cell types. By attaching ligands or antibodies that can bind to markers specific to TB-infected bone tissue, PLGA nanoparticles can be directed more precisely to the site of infection, potentially increasing the treatment’s efficacy and reducing off-target effects [122,123,124,125]. A notable application of PLGA in bone TB treatments is demonstrated in a study where a nanoscale delivery system consisting of INH-conjugated PLGA (PLGA-INH4) combined with beta-tricalcium phosphate (b-TCP) was developed [90]. This innovative composite scaffold system was engineered to control the release of INH for over 100 days, achieving high localized drug concentrations and minimal systemic exposure. Implemented in an in vivo model, this system involved implanting the scaffold into artificial bone defects in New Zealand rabbits. The approach yielded significant advancements in bone TB treatment, including sustained effective INH levels and the facilitation of bone regeneration, while potentially minimizing the systemic side effects typically associated with TB medications.

### 4.5. Bovine Serum Albumin Nanoparticles

Bovine serum albumin (BSA) nanoparticles are at the forefront of biomedical research, offering significant advantages in drug delivery due to their biocompatibility, biodegradability, and ease of modification [126,127]. Derived from a readily available and cost-effective protein source, BSA nanoparticles excel in encapsulating a wide array of therapeutic agents, ranging from small molecule drugs to proteins and nucleic acids. This capability enhances the solubility and stability of encapsulated drugs, potentially reducing side effects and improving therapeutic efficacy. A key feature of these nanoparticles is the ability to modify their surface with targeting ligands, enabling the selective targeting of specific cells or tissues and increasing the precision of drug delivery. BSA nanoparticles are known for their favorable safety profile, making them suitable for various clinical applications. Their typical size range of 10 to 200 nanometers facilitates efficient cellular uptake and effective delivery to targeted sites while also allowing them to evade rapid immune clearance, prolonging their circulation time in the body [128,129].

In a groundbreaking study by Ma et al. [27], BSA nanoparticles were used to deliver INH and RFP intravenously for the treatment of spinal TB in a rabbit model. This innovative approach showed marked improvements in targeting the affected spinal regions in vivo. Characterized by an average size of 60.5 ± 4.6 nm and high encapsulation efficiency, the nanoparticles enabled a sustained drug release directly at the infection site. Therapeutic efficacy was demonstrated by significant bone tissue repair and necrotic tissue replacement with normal bone, as evidenced by CT scans and histological examinations after 6 and 12 weeks of treatment. The use of this nanoscale delivery system notably reduced the frequency of drug administration, thereby diminishing the potential for adverse effects commonly associated with systemic drug exposure, such as toxicity to the liver, spleen, and lungs. Moreover, this approach not only enhanced the local drug concentration at the TB infection site but also alleviated neurological symptoms, such as hind leg paralysis, which were observed in the traditional drug administration group.

### 4.6. Nanoscale Mineralized Collagen

Nanoscale mineralized collagen, a sophisticated biomaterial, is ingeniously designed to emulate the complex nanostructure and composition of natural bone. This innovative composite integrates nano-hydroxyapatite (nHA) [130,131,132], akin to bone’s inorganic mineral phase with type I collagen.

Hydroxyapatite (HA), chemically denoted as Ca_10_(PO_4_)_6_(OH)_2_, is a naturally occurring mineral form of calcium apatite with hydroxide ions. It is a key constituent of bone, making up the hard matrix that gives bone its rigidity and strength. Due to its significant similarity to natural bone minerals, HA is extensively studied and utilized in biomedical applications, particularly in bone tissue engineering and regenerative medicine [133,134,135]. The structure of HA is characterized by its high biocompatibility and osteoconductivity, which facilitate its integration into bone tissue. These properties make HA an ideal material for scaffolds in bone regeneration applications. HA scaffolds are porous structures designed to mimic the natural architecture of bones, providing a framework for new bone growth and development. The porosity and interconnected pore structure of these scaffolds are crucial, as they allow for cell infiltration, nutrient transfer, and waste removal [136,137,138]. In the context of treating bone TB, HA scaffolds offer a potential platform for bone reconstruction and drug delivery. Bone TB often leads to the destruction of bone tissue, necessitating interventions that can support bone regeneration. HA scaffolds can be used to fill bone defects caused by TB, providing a matrix for new bone growth and helping restore the structural integrity of affected bones. Additionally, HA’s compatibility with bone tissue makes it an attractive candidate for localized drug delivery systems [139,140]. The potential to incorporate anti-tubercular drugs into HA scaffolds presents an innovative approach to TB treatments. By embedding drugs directly into the scaffold, localized drug delivery can be achieved at the site of the bone TB infection. This method can provide a sustained release of medication, maintaining therapeutic drug levels directly at the infection site, which is crucial for effectively treating TB given the slow replication rate of Mycobacterium TB and the typically prolonged treatment regimens [74,84,141,142]. Furthermore, the customization of HA scaffolds allows for the tailoring of pore size and porosity to match the specific requirements of the bone defect and the desired drug release profile. However, challenges in the development of HA scaffolds for bone TB treatments include optimizing the drug-loading capacity, releasing kinetics, and ensuring the mechanical strength of the scaffold to support bone regeneration while delivering the therapeutic agents effectively [31,143,144,145].

Type I collagen, a primary structural protein found abundantly in various connective tissues, plays a pivotal role in tissue engineering and regenerative medicine. It is a fibrous protein that constitutes the majority of the extracellular matrix in bones, providing structural integrity and flexibility [146,147,148]. This collagen type is characterized by its unique triple-helix structure, contributing to its mechanical strength and stability. Its biocompatibility and ability to promote cell attachment and proliferation make it an ideal scaffold material in tissue regeneration. Moreover, its natural abundance and ease of modification enhance its suitability for diverse biomedical applications, from wound healing to bone tissue engineering [149,150]. This strategic amalgamation not only replicates the bone’s hierarchical architecture but also fosters an environment conducive to cellular adhesion and proliferation. The intricately tailored porosity and surface characteristics of this material are pivotal in enhancing osteoconductivity and osteoinductivity, crucial elements in bone regeneration and repair. Additionally, its intrinsic capability to encapsulate therapeutic agents such as isoniazid positions it at the forefront of targeted drug delivery systems, specifically for bone-related pathologies.

In one study [92], a nanoscale drug delivery system called isoniazid-loaded mineralized collagen scaffold was developed for bone tuberculosis treatment. This system, assessed through an in vivo mouse model, effectively delivered isoniazid directly to the affected bone areas, thereby demonstrating potential in targeting and treating bone tuberculosis. The sustained release of isoniazid from the nanoscale system reduced the likelihood of systemic side effects, a common issue with traditional tuberculosis treatment methods. Compared to traditional drug delivery systems, this implant offers enhanced drug release performance, biodegradability, and biocompatibility, showing significant potential for tuberculous bone and joint repair applications. This approach epitomizes the synergy between material science and regenerative medicine, spotlighting the potential of such biomimetic strategies in pioneering new frontiers in bone repair therapies and advanced drug delivery mechanisms.

### 4.7. Chitosan/Carbon Nanotubes Nanoparticles

Chitosan (CS), a biopolymer derived from chitin in crustacean shells, stands out in nanoscale drug delivery due to its unique chemical and biological properties. Its structure, consisting of glucosamine and N-acetylglucosamine linked by β-(1-4)-glycosidic bonds, imparts key characteristics [151,152,153]. The cationic nature of CS under acidic conditions facilitates the formation of nanoparticles through ionic gelation with negatively charged molecules, enhancing drug encapsulation efficiency. Its biocompatibility and biodegradability ensure minimal toxicity and side effects in biomedical applications [154,155]. CS‘s mucoadhesive property improves mucosal drug absorption, while its ability to transiently open tight junctions in epithelial cells enhances permeability and targeted drug delivery.

Carbon nanotubes (CTNs), cylindrical nanostructures composed of rolled-up sheets of graphene, are renowned for their exceptional mechanical strength, electrical conductivity, and thermal stability. In the realm of drug delivery, their high aspect ratio and large surface area make them excellent carriers, allowing for a high drug-loading capacity. The surface of CNTs can be chemically modified to improve solubility and biocompatibility. Their ability to penetrate cell membranes facilitates efficient drug delivery to targeted sites, making them promising tools in cancer therapy and other treatments where targeted action is critical.

In one study, Chen et al. [91] utilized a nanoscale delivery system comprising CS/CTN nanotube nanoparticles to encapsulate isoniazid. A guinea pig model of tuberculous ulcer was built to simulate the clinical scenario of bone tuberculosis. This nanoscale delivery system demonstrated significant positive effects in the treatment model. It not only promoted the healing of tuberculosis ulcers, evidenced by a notable reduction in the ulcer area (94.6% decrease compared with the isoniazid group and 89.8% decrease compared to the isoniazid/CTN group) but also effectively reduced the number of Mycobacterium tuberculosis at the ulcer sites. Additionally, the CS/CTN nanoparticles significantly lowered the cytotoxicity and inflammatory reaction induced by isoniazid, as indicated by the reduced apoptosis rate and CD3þT cells and CD4/CD8þ cell ratio.

Xie et al. [93]. developed an innovative nanostructured delivery system, the n-HP@ICG scaffold, for the targeted administration of INH. This composite scaffold was synthesized by attaching INH to CS and crosslinking them using glutaraldehyde to a porous nanohydroxyapatite/polyamide 66 (n-HA/PA66) framework. Intricately engineered, the scaffold effectively harnessed the biocompatibility of chitosan for drug loading and a controlled release. In vitro tests showed a sustained drug release over approximately 15 days, while in vivo experiments, conducted using a rabbit femoral condyle defect model, indicated a prolonged drug release of up to 28 days. This innovative approach substantially enhanced osteoarticular tuberculosis treatments by maintaining an effective drug concentration at the bone lesion site. Additionally, it significantly reduced potential side effects, such as liver and kidney toxicity, commonly associated with systemic tuberculosis medication administration. The n-HP@ICG scaffold effectively inhibited the activity, proliferation, and adhesion of Mycobacterium tuberculosis while also maintaining satisfactory osteoconduction and osseointegration.

## 5. Conclusions, Challenges, and Future Outlook

The exploration of nanoscale drug delivery systems (NDDS) in the treatment of bone TB represents a pivotal advance, addressing inherent challenges in current therapeutic strategies. Traditional treatments often face difficulties in effectively targeting the infection, constrained by drug specificity and systemic toxicity. NDDS, including liposomes, mesoporous silica nanoparticles, tetracycline-modified nanoparticles, and others, offer a groundbreaking solution by ensuring precisely targeting infection sites and controlling the drug release. These systems improve drug bioavailability, delivering higher concentrations of therapeutic agents directly to affected bone areas. This targeted approach enhances therapeutic efficacy and significantly reduces adverse effects associated with conventional TB treatments. In the realm of bone tuberculosis treatments, nanocarriers, particularly mesoporous silica nanoparticles, show great potential for practical applications due to their distinct structural properties. These nanoparticles are characterized by an extensive surface area and significant porosity, which are instrumental in their ability to encapsulate substantial quantities of therapeutic agents. Such a high drug-loading capacity is vital for the efficient delivery of medications, a necessity in conditions, such as bone tuberculosis, that demand targeted and prolonged drug release. Furthermore, the adjustable pore size and surface chemistry of these nanoparticles can be finely tuned, providing a mechanism to control drug release kinetics and enhance therapeutic outcomes. Additionally, their inherent biocompatibility and the potential for surface modifications to target specific sites significantly bolster their clinical applicability.

In the complex scenario of bone TB, characterized by intricate structures and granulomatous inflammation, NDDS’s precision can lead to more effective treatment outcomes. Their adaptability to specific stimuli within the bone TB microenvironment, such as pH changes, allows for a controlled and sustained drug release, thereby enhancing treatment effectiveness and reducing harm to healthy tissues. The capability of NDDS to encapsulate drugs and prevent their premature degradation further amplifies their effectiveness, and these systems also have the potential for personalized treatment, considering the variable nature of bone TB and its diverse responses to therapy.

However, despite the promising potential of NDDS in bone TB treatments, challenges remain. These include the need for comprehensive long-term safety evaluations, the potential emergence of drug resistance, scalability challenges, and complexities in manufacturing. The translation from bench to bedside is complex, and clinical successes may not always mirror pre-clinical findings, emphasizing the unpredictability of clinical applications. The field of NDDS in bone TB treatments is still evolving, with a significant lack of extensive clinical trials and related data. This gap underscores the importance of continued research and innovation, particularly in translating these advanced therapeutic strategies into clinical practice.

At the same time, while our review has highlighted several key advancements in nanotechnology for bone TB treatments, it is important to acknowledge that this field is still in its early stages. The limited number of studies identified and reviewed herein reflects the emerging nature of this research area. This nascent stage is characterized by pioneering exploratory studies and a growing understanding of the potential applications of nanotechnology in bone TB.

Moreover, the field still needs to address unexplored territories, such as the long-term in vivo behavior of nanoparticles, their systemic impacts, and interactions within the complex bone TB pathology. Future research should focus on developing more biocompatible and biodegradable nanoparticles, investigating targeted delivery systems for multidrug-resistant TB strains, and conducting large-scale clinical trials to confirm the efficacy and safety of these nanosystems.

A key area for future exploration involves the integration of artificial intelligence (AI) and machine learning (ML) technologies. AI and ML can play a transformative role in the design and optimization of NDDS, offering powerful tools for predicting nanoparticle behavior, optimizing drug delivery mechanisms, and personalizing treatments based on patient-specific data. These technologies can significantly accelerate the development of NDDS by analyzing vast datasets to identify patterns and optimize nanoparticle properties for enhanced efficacy and safety.

A multidisciplinary collaboration involving experts in nanotechnology, pharmacology, TB treatment, and AI/ML is crucial for achieving breakthroughs in this field. This review lays the groundwork for future exploration and development in NDDS for bone TB, highlighting the transformative potential of these nanotechnologies in TB treatments. It presents a potential paradigm shift in therapeutic strategies for bone TB, showcasing the promise of enhancing drug bioavailability, reducing adverse effects, and providing a more targeted approach. However, translating these promising preclinical findings into clinical practice requires addressing challenges in scalability, safety, and regulatory approval. This review paves the way for future advancements in this exciting field, aiming to transition these therapies from bench to bedside and offering new hope for patients with bone TB.

## Figures and Tables

**Table 1:** First-line anti-tuberculosis medications.

Drug	Dosage	Duration	Mechanism of Action	Advantages	Disadvantages
Isoniazid	5 mg/kg/day (max 300 mg/day)	6–18 months	Inhibits mycolic acid synthesis, essential for cell wall	Highly potent, good penetration into tissues	Hepatotoxicity, Peripheral neuropathy
Rifampicin	10–20 mg/kg/day (max 600 mg/day)	6–18 months	Inhibits DNA-dependent RNA polymerase, blocking RNA synthesis	Broad activity, reduces treatment duration	Hepatotoxicity, Drug interactions
Pyrazinamide	15–30 mg/kg/day (max 2 g/day)	Initial 2 months	Effective in acidic pH, disrupts mycobacterial cell membrane	Effective against latent TB	Hepatotoxicity, Hyperuricemia
Ethambutol	15–25 mg/kg/day	Initial 2 months	Inhibits arabinose transferase, affecting cell wall biosynthesis	Prevents resistance emergence	Optic neuritis, Visual disturbances

**Table 2:** Bone graft materials of reconstruction for bone tuberculosis.

Material Type	Source	Properties	Clinical Use	Advantages	Disadvantages	References No.
Autografts	Patient’s own bone	Osteogenic, osteoinductive, osteoconductive, biocompatible	Preferred for its biological properties	Best integration and growth potential	Limited availability, donor site complications	[76]
Allografts	Donor human bone	Various forms, may be processed	Useful when autograft quantity is insufficient	Reduced donor site morbidity	Risk of disease transmission, immune response	[76]
Xenografts	Bone from another species	Processed for biocompatibility	Alternative when human bone is not preferred	No risk of disease transmission from human	Cross-species compatibility issues	[77]
Bone Graft Substitutes	Synthetic or naturally derived	Includes ceramics, cements, glass	Fill bone defects and provide a scaffold	Variety of options and ease of use	Lack of osteogenic, osteoinductive, osteoconductive, and biocompatible properties	[76]
Vascularized Bone Grafts	Bone with its own blood supply	Improved healing in avascular areas	Used in challenging defects	Superior in areas with poor blood supply	Technically demanding, donor site morbidity	[78]
Custom 3D-Printed Implants	Based on patient-specific imaging	Tailor-made, biocompatible	Perfect fit for defect area	Custom-fit, reduces adaptation issues	High-cost, complex pre-surgical planning	[79]
Titanium Mesh	Metallic scaffolds	Support and allows bone growth	Spinal fusion surgeries	Immediate structural support	Biological incompatibility, subsidence, stress shielding, and radiopacity	[80,81]
Polymers	Biodegradable or non-biodegradable	Scaffolds for bone regeneration	Gradual bone regeneration	Versatility and controlled degradation	May induce inflammatory response	[82]
Metal Alloys	Stainless steel, cobalt-chromium, etc.	Used for structural support	Load-bearing area repair	High strength and fatigue resistance	Stress shielding, toxic ion release, secondary surgery, and imaging artifacts	[83]

**Table 3:** The summary of nanocarriers designed for bone tuberculosis.

Author/ Year	Types of Delivery System	Drug Loaded	Animal Model	Designed Route of Administration	Target Area	Release Time	Effect	Limitation	References No.
Zhu et al., 2015	Mesoporous Silica NPs	IHN/RIF	New Zealand rabbits	Implantation into rabbit femoral bone defects	Bone tuberculosis foci	84 days	Direct drug delivery to bone TB sites promotes bone growth and limits side effects on the liver and kidneys	Faces challenges with biodegradability and stability, including potential pore blockage and surface modifier degradation	[30]
Zhu et al., 2011	Mesoporous Silica NPs	IHN/RIF	New Zealand rabbits	Implantation into rabbit femoral bone defects	Bone tuberculosis foci	30 days	Ensuring prolonged drug efficacy while minimizing systemic side effects	Faces challenges with biodegradability and stability, including potential pore blockage and surface modifier degradation	[29]
Yahia et al., 2023	Mesoporous Silica NPs	LVX/RIF	Wistar rats	Subcutaneous implantable composite scaffold	Bone tuberculosis foci	60 days	Lowers drug IC50, aiding in spinal repair and regeneration, with minimal biological side effects	Similar biodegradability and clearance issues; may encounter pore clogging and surface alteration	[89]
Yahia et al., 2023	Mesoporous Silica NPs	LVX/RIF	Wistar rats	Subcutaneous implantable composite scaffold	Bone tuberculosis foci	30 days/32 days	Delivers TB medication directly to infection sites for sustained effect, reducing systemic drug dependency	Similar biodegradability and clearance issues; may encounter pore clogging and surface alteration	[32]
Liang et al., 2023	Tetracycline-modified NPs	RPT	Kunming mice	Vein injection	Bone tuberculosis foci	60 h	Increases rifapentine’s efficacy in osteoarticular TB, minimizing dosage and treatment frequency	May promote the development of resistance in bacteria	[28]
Huang et al., 2015	Poly(lactide-co-glycolide) NPs	IHN	New Zealand rabbits	Implantation into rabbit radius bone defects	Bone tuberculosis foci	100 days	Achieves long-term, localized drug release and facilitates bone healing	Water-soluble drugs face integration challenges; degradation byproducts may affect drug release and tissue health	[90]
Ma et al., 2021	Bovine serum albumin NPs	IHN/RIF	New Zealand rabbits	Vein injection	Bone tuberculosis foci through systemic circulation	42 days	Continuous drug release at the infection site enhances treatment and lowers adverse reactions	Risks immunogenic reactions; variable composition may affect consistency and safety	[27]
Liu et al., 2019	Liposome NPs	DINH	New Zealand rabbits	Intra-articular injection	Bone tuberculosis foci	72 h	Provides stable drug levels at the infection site, potentially decreasing dosing frequency and reducing side effects	Susceptible to oxidation and hydrolysis; may have limitations in carrying hydrophobic drugs	[85]
Chen et al., 2019	Chitosan/carbon nanotubes NPs	INH	Guinea pigs	Vein injection	Secondary wound of bone tuberculosis through systemic circulation	48 h	Supports ulcer healing and reduces bacterial load and isoniazid-induced toxicity	Toxicity and immunogenicity are concerns; non-biodegradability poses environmental risks	[91]
Fang et al., 2022	Nanoscale mineralized collagen	INH	Kunming mice	Subcutaneous implantable composite scaffold	Bone tuberculosis foci	84 days	Delivers isoniazid effectively to bone, with improved biodegradability and compatibility	Collagen’s variability can lead to inconsistent properties and potential immunogenicity	[92]
Xie et al., 2021	Chitosan NPs	INH	New Zealand rabbits	Implantation into rabbit femoral bone defects	Bone tuberculosis foci	28 days	Inhibits TB bacteria growth and adhesion, promoting bone integration and health	Risk of immunogenicity and allergic reactions; may aggregate in biological fluids	[93]

Abbreviations: NPs, nanoparticles; INH, isoniazid; RIF, rifampicin; LVX, levofloxacin; DINH, N′-Dodecanoylisonicotinohydrazide; RPT, Rifapentine.

## Data Availability

No new data were created or analyzed in this study. Data sharing is not applicable to this article.
